# Reducing motion sickness during simulated astronaut post-spaceflight water landings using anticipatory cues or postural control

**DOI:** 10.1038/s41526-025-00478-9

**Published:** 2025-06-02

**Authors:** Taylor L. Lonner, Aaron R. Allred, Aadhit R. Gopinath, Tori Morgheim, Eric L. Groen, Charles M. Oman, Paul DiZio, Ben D. Lawson, Saige R. Drecksler, Torin K. Clark

**Affiliations:** 1 Smead Department of Aerospace Engineering Sciences, University of Colorado-Boulder, Boulder, CO USA; 2 Human Performance Department, TNO, Soesterberg, The Netherlands; 3 Human Systems Laboratory, Massachusetts Institute of Technology, Cambridge, MA USA; 4 Ashton Graybiel Spatial Orientation Laboratory, Brandeis University, Waltham, MA USA; 5 Volen Center for Complex Systems, Brandeis University, Waltham, MA USA; 6 Psychology Department, Brandeis University, Waltham, MA USA; 7 Naval Submarine Medical Research Laboratory, Groton, CT USA

**Keywords:** Neuroscience, Physiology, Engineering

## Abstract

Astronauts returning to Earth after adapting to microgravity are susceptible to Entry Motion Sickness while they are readapting to 1G. We assessed the efficacy of two countermeasures in reducing the incidence and severity of motion sickness symptoms using a series of ground-based analogs meant to simulate the scenario of a post-spaceflight water landing: one hour of habituation to 2Gx centrifugation followed by up to an hour of passive wave-like motion at 1 G. The first countermeasure provided rich visual cues of current self-motion overlaid with anticipatory cues of self-motion one second in the future, presented in virtual reality with the subject’s head and torso restrained. The second countermeasure encouraged active postural control by instructing subjects to keep their unrestrained head aligned with Earth-vertical during wave-like motion. Both groups were compared to a control group that did not receive any Earth-fixed visual cues and had the head and torso restrained. As a secondary metric, we also considered how these countermeasures impacted vestibular-mediated standing balance performance. While the multi-symptom Motion Sickness Questionnaire scores did not significantly differ between the three groups, the development of gastrointestinal symptoms was diminished for the anticipatory visual cues group compared to the control ($p=0.03$) and active posture ($p=0.02$) groups. Additionally, the anticipatory cues group was significantly more likely to tolerate the full period of wave-like motion (90% of subjects with cues vs. 33% without, $p=0.017$). Finally, across all three groups, subjects had significantly increased sway ($p=0.0002$) following wave-like motion, which returned to a baseline equivalency after an hour of recovery. Enabling the brain to form a better expectation of sensory stimulation, anticipatory cues reduce the incidence of nausea, which may be beneficial for motion sickness in astronauts, as well as here on Earth.

## Introduction

Historically, transitions to a novel gravity environment have elicited a series of biomechanical and vestibular-mediated deficits in astronauts. One such problem, motion sickness, is characterized by varied symptomology induced by real or apparent motion^1^, and can manifest in humans as severe discomfort, nausea, and/or vomiting. During transit to or from space, motion sickness occurs concurrently with incapacitation or degraded cognitive and sensorimotor performance, often exacerbated by head movement-contingent vertigo^2,3^, which collectively constitute operational risks during spaceflight and are driving factors for delaying extravehicular activities (space walks)^4,5^.

An estimated 60-80% of the astronaut population^6,7^ experiences space adaptation syndrome or ‘space motion sickness’ (SMS), a condition that occurs when humans undergo a transition into microgravity and are transiently maladapted. The most severe symptoms of SMS occur within the first 72 hours following the transition to microgravity, but symptoms sometimes continue for up to a week^8,9^. Following this adaptation to microgravity, astronauts returning to Earth experience a reciprocal maladaptation following re-entry coined ‘entry motion sickness’ (EMS)^7,10^ or ‘terrestrial readaptation motion sickness’ (TRMS)^11,12^ more recently, which includes motion sickness (e.g., nausea) and sensorimotor coordination problems (e.g., imbalance). EMS symptoms are similar in progression to SMS symptoms and are likely to be exacerbated by bouts of active motion as well as by sustained passive motion, such as the potentially substantial sea state motion during water landings (as performed with the SpaceX Dragon and NASA Orion capsules). As such, EMS poses operational risks in terms of incapacitating astronauts returning to Earth, in addition to the discomfort associated with nausea. In a capsule undergoing a water landing, EMS causes additional hazards should an emergency occur, such as unexpected sea state motions, recovery delays, or capsule damage or malfunction, requiring the astronauts to perform mitigating operations or to egress prior to recovery.

It is believed that both SMS and EMS arise due to excessive, unexpected sensory information from the graviceptors due to gravity transitions. For example, the otoliths within the vestibular system are affected only by translational acceleration in microgravity, instead of the combination of translational acceleration and acceleration due to Earth’s gravity^13^. This novel information generates sensory conflicts between neural expectations associated with past movements in 1 G versus the peripheral sensory afference experienced in 0 G and vice versa, activating motion sickness pathways within the brain^14–16^, as evidenced by neural correlates in both the brainstem and cerebellum^17^. Pharmaceuticals (e.g., promethazine or scopolamine in microgravity^4^, or meclizine prior to re-entry and landing^18^) have been used^19–23^, but have undesirable side effects and concerns regarding shelf-life and stability.

To avoid the limitations associated with pharmaceuticals and improve our basic understanding of factors modulating motion sickness, there exists a need to develop non-pharmacological countermeasures for SMS and EMS. In the terrestrial environment, the essential role of sensory expectations with regard to motion sickness can be explored by considering the driver of a car. The experienced driver can form an expectation of upcoming sensory signals as they make active control inputs to the pedals and steering wheel, helping minimize sensory conflict such that drivers rarely experience carsickness^24–26^. For experienced sailors, anecdotal evidence has suggested that engaging in active postural control operations such as “wave riding,” reduces seasickness^27^. The process of swaying the body to counteract the rocking of the ship also reduces the peripheral vestibular stimulus caused by the ship motion and allows the CNS to produce a better expectation of the incoming sensory signals.

In addition to active control, having information about upcoming self-motion (i.e., anticipatory sensory cues) can also allow the CNS to produce appropriate expectations. The passenger in the front seat of a vehicle may not have control over the vehicle, but being able to see the road ahead allows them to generate better expectations of both present and future motion sensations compared to a rear-seat passenger who experiences a predominantly internal and self-fixed visual scene^28,29^. For back seat passengers, multiple studies have been performed to analyze the efficacy of anticipatory cues of upcoming motion in reducing motion sickness. Visual anticipatory cues have been generated using peripheral LED light displays^30,31^, stylistic rollercoaster tracks^32^, and virtual reality scenes^25^, and have shown promise in reducing motion sickness severity. Auditory^33–35^ and vibrotactile^36–38^ displays have also been used to cue upcoming motions with moderate success in mitigating motion sickness.

When exposed to varying, passive wave motion within a capsule floating at sea, an astronaut’s CNS has limited means to formulate accurate expectations of the incoming sensory signals. However, expectations can be generated either using active control – as done by the experienced vehicle driver – or by generating anticipatory cues of future motion – as done by the front seat passenger. Using these concepts, we can “engineer” sensory cues that reduce sensory conflict. This study investigates these approaches for the application of post-spaceflight water landings, quantifying the incidence and severity of motion sickness during a ground-based analog requiring sensorimotor adaptations after G transitions. To simulate the gravity transition aspect of the post-spaceflight environment, Sickness Induced by Centrifugation (SIC) was employed^39^, exposing subjects to one hour of 2Gx hyper-gravity before returning to normal gravity, to recreate symptoms of motion sickness following gravity transitions. Sea state was simulated using a roll-tilt and lateral translation profile generated from frequency sampling the movement of a Pacific Ocean buoy.

Primarily, we hypothesized that, following a gravity transition, subjects who either used active postural control (tasked with using their neck and torso to keep their head upright with Earth gravity) or received visual cues including anticipatory cues of their upcoming motion one second in the future would experience less motion sickness during simulated wave-like motion compared to a control condition that received neither intervention. Secondarily, using balance as a proxy for sensorimotor performance, we hypothesized that subjects who received either of the countermeasure treatments during the wave-like motion would have better vestibular-mediated balance performance compared to the control condition. This work is a continuation of Lonner et al.^40^ which investigated the benefits of non-anticipatory visual cues.

## Results

Well-established and reliable subjective ratings for motion sickness and anxiety were assessed at five-minute increments during wave-like motion using the Motion Sickness Questionnaire (MSQ)^41,42^ and the modified State-Trait Anxiety Inventory (STAI)^43^, respectively. Nausea was separately assessed every minute on a scale of ‘none’, ‘slight’, ‘moderate’, and ‘severe’. All subjects wore an Oculus Quest 2 virtual reality headset as the countermeasure or control delivery mechanism. Only subjects in the active posture experimental group had their heads and torsos unrestrained. These and other methods are detailed in a subsequent section.

### Motion Sickness and Anxiety

**Fig. 1:**
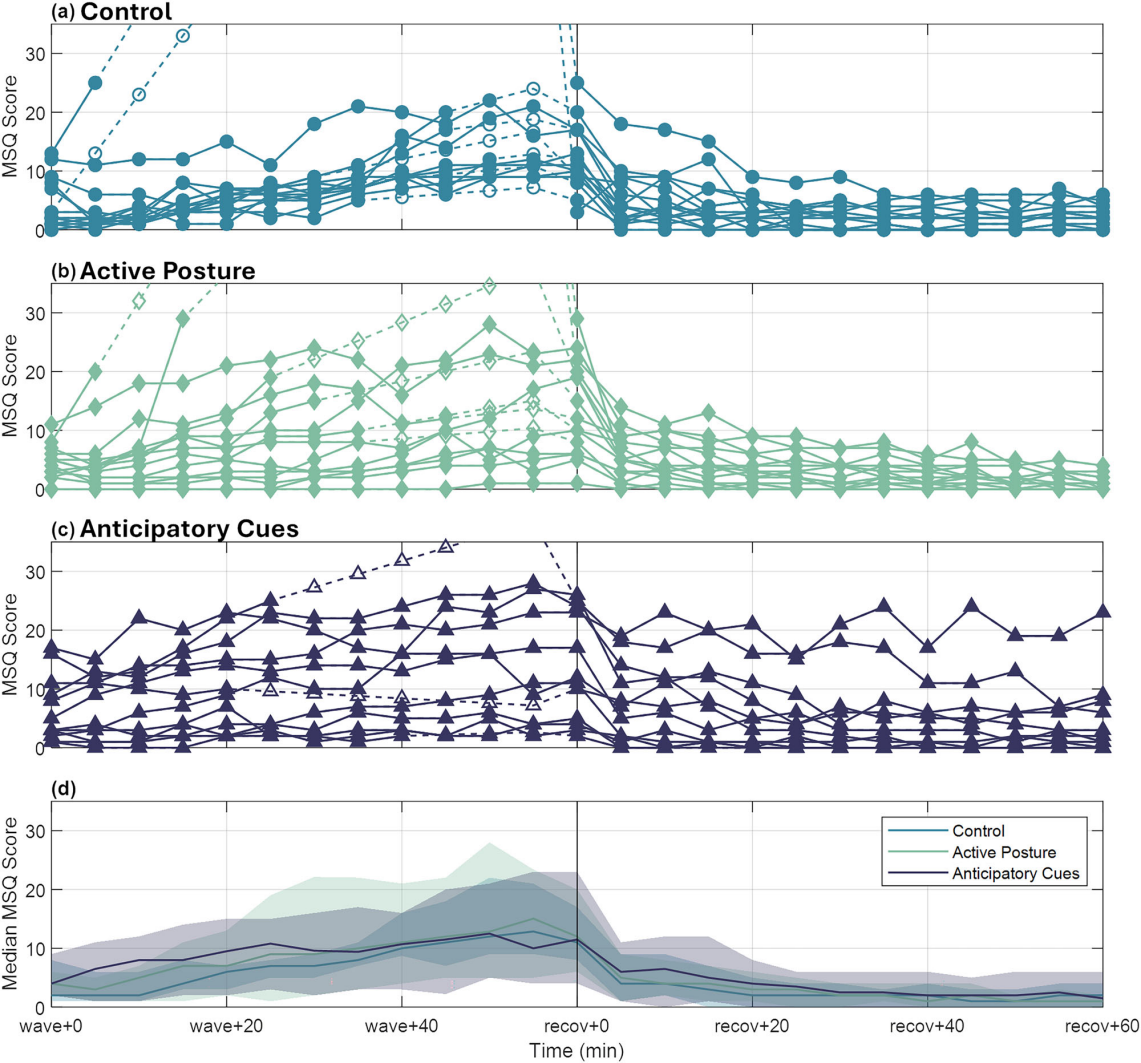
Time progression of Motion Sickness Questionnaire scores. Individual scores for the **a** control group, **b** active posture group, and **c** anticipatory cues group are presented. The bottom subplot (**d**) shows the overlay of median and quartile ranges for all three groups. Filled shapes and solid lines represent actual data points while open shapes and dashed lines represent estimates after a subject dropped out by extrapolation of a linear fit to that subject’s data prior to dropout.

The descriptive time progression of MSQ scores for all three experimental groups is shown in Fig. 1. Individual scores for the control, active posture, and anticipatory cues groups are shown in the top three subplots while Fig. 1d shows the median score for each group, including linearly extrapolated data when subjects dropped out due to excess nausea (i.e., more than two consecutive minutes of ‘moderate’ nausea), as well as the quartile ranges. In general, MSQ scores gradually increased during wave-like motion and subsided quickly once the wave-like motion stimulus ended. Despite the similar medians at each time point, and the substantial overlap in quartile ranges between the three experimental groups, looking more closely at symptom subcategories, there were some apparent differences between groups.

**Fig. 2:**
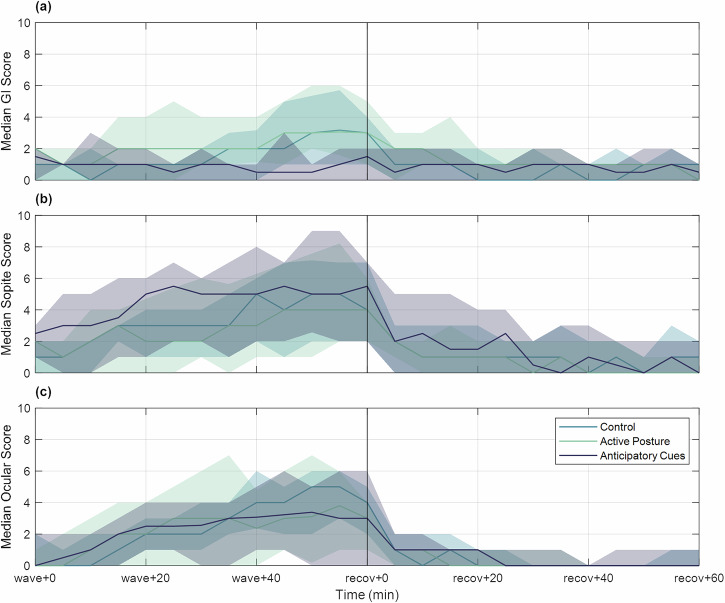
Time progression of Motion Sickness Questionnaire subcategory scores. Median and quartile ranges are shown for all three experimental groups for **a** gastrointestinal disturbance symptoms, **b** sopite syndrome symptoms, and **c** ocular symptoms.

Breaking the 28-symptom MSQ reports into subcategories based on the work of Cha et al.^44^, we identified that gastrointestinal disturbance symptoms between the control and active posture groups had a similar progression and recovery, but the anticipatory cues group had notably less gastrointestinal disturbance progression (Fig. 2a) with the median line remaining mostly flat through wave-like motion and recovery. Similarly, for sopite-relevant symptoms^45,46^ from the MSQ (encompassing general apathy and malaise), the control and active posture groups had a comparable time course of development, while the anticipatory cues group appeared to begin with a higher score but plateaued around the ‘wave+30’ time point rather than continuing to increase (Fig. 2b). Ocular symptoms (those hypothesized to be exacerbated by the presence of the VR headset)^47^ progressed similarly across all three experimental groups (Fig. 2c).

**Fig. 3:**
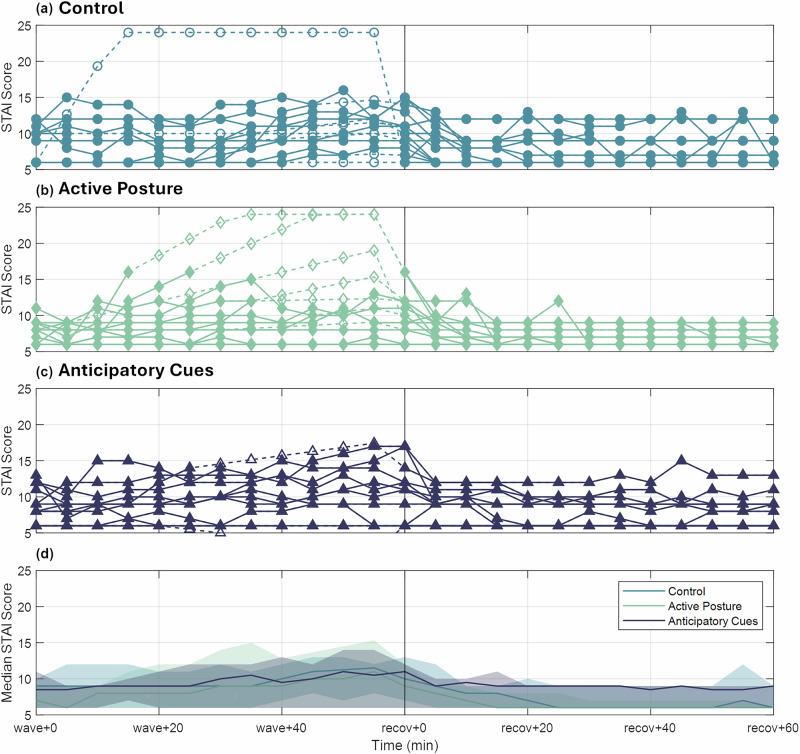
Time progression of STAI scores. Individual scores for the **a** control group, **b** active posture group, and **c** anticipatory cueing group are presented. Median values and quartile ranges are shown in (**d**).

Anxiety was also monitored during wave-like motion using a modified STAI (Fig. 3). In general, there was a slight increase in anxiety during wave-like motion, followed by a return to baseline during recovery, but there were no notable differences between the three experimental groups.

Having qualitatively compared the time course of motion sickness symptom development, we performed statistical comparisons, focusing on the effect of the three experimental groups (control group, active posture, and anticipatory cues). In each case, we assessed the impact of the experimental group and included potential covariates (i.e., participant’s past history of motion sickness in other situations via the Short-Form Motion Sickness Susceptibility Questionnaire (MSSQ)^48,49^, reported STAI, or participant sex) when it helped reduce the variance in subject groups, enabling a more precise test of experimental group (only the final, most precise model is presented in each case). First, we compared MSQ scores at the end of the wave-like motion (wave+55) when motion sickness was most severe. Second, we compared MSQ scores half-way through our wave-like motion (wave+30), which may represent a ”typical” capsule recovery period based upon recent SpaceX Crewed Dragon recoveries to date^50^. Third, we considered the linear “slope” of the MSQ reports over time to capture the progression of motion sickness. In each of these analyses, we included various covariate predictors (participant MSSQ, participant sex, and/or reported STAI at the same time point) to account for variance associated with these uncontrolled factors.

Given the non-parametric nature of the MSQ scores (ordinal rating of “none”, “slight”, “moderate” or “severe” that are summed), cumulative link models were fitted to MSQ scores at ‘wave+30’ and ‘wave+55’. While the primary independent variable was the experimental group, we also included each participant’s Motion Sickness Susceptibility (MSSQ) percentile, and currently reported STAI score as covariate predictors (MSQ ~ Group + MSSQ + STAI). The experimental group was not observed to be significant at either time point (Active Posture: ${\beta }_{{wave}+30}=-0.42,{p}_{{wave}+30}=0.53;{\beta }_{{wave}+55}=-0.51,{p}_{{wave}+55}=0.45$. Anticipatory Cues: ${\beta }_{{wave}+30}=-0.42,{p}_{{wave}+30}=0.55;{\beta }_{{wave}+55}=-0.79,{p}_{{wave}+55}=0.28$). At both time points, STAI was found to be a significant covariate (${\beta }_{{wave}+30}=0.43,{p}_{{wave}+30}=5.2\times {10}^{-7};{\beta }_{{wave}+55}=0.37,{p}_{{wave}+55}=2.1\times {10}^{-6}$). Additionally, participant MSSQ was found to be a significant covariate at the 30-minute mark during wave-like motion ($\beta =0.024,p=0.028$). Motivated by the relationship between MSQ and STAI scores, a Spearman correlation test was performed between the two metrics. Pooling subject reports across the ‘wave+30’ and ‘wave+55’ time points, a significant positive correlation was found ($\rho =0.73,p=2.2\times {10}^{-16}$).

Additional cumulative link models were fitted to the subcategory scores at the same time points to determine if experimental group, MSSQ, or anxiety had any impact on specific symptom groups within the MSQ (MSQ_subcat_ ~ Group + MSSQ + STAI). The effect of the anticipatory cues group on GI symptoms was a near-significant trend, at ‘wave+55’ ($\beta =-1.38,p=0.06$), while no other subcategories revealed detectable significant effects of anticipatory cues or active posture at either ‘wave+30’ or ‘wave+55’ ($p\in [0.14,\,0.95]$). Again, a significant correlation to STAI was found for all subcategory scores at both time points ($p\in [{10}^{-5},0.004]$), and the effect of MSSQ on GI symptoms was significant at ‘wave+30’ $(p\in[0.14, 0.95])$. This implies that higher MSSQ percentiles result in higher GI scores earlier in the wave-like motion, but the anticipatory cues were effective at alleviating this effect on GI scores following 55 minutes of wave-like motion.

To supplement the previous analysis with a more time-dependent metric, a linear slope was fitted to the time series of the MSQ scores for each subject to capture motion sickness progression. This was used as a dependent variable for an aligned rank transform analysis of variance with experimental group, sex, and MSSQ level as predictors.

The aligned rank transform of the full MSQ slope revealed a significant effect of the experimental group ($F(2,30)=3.82,p=0.03$), as well as significance of participant sex ($F(1,30)=6.70,p=0.01$) and an interaction between sex and MSSQ level ($F(1,30)=5.63,p=0.02$). A post-hoc Kruskal-Wallis test did not detect a relationship between MSQ slope and experimental group, but a post-hoc Wilcoxon Rank Sum test did find that female subjects had a higher MSQ slope than male subjects ($W=305,p=0.03$), corresponding to a faster rate of motion sickness development in females, with a medium effect size according to the probability of superiority ($f=0.70$).

Since the cumulative link models found a trend between GI scores and experimental group, an aligned rank transform analysis of variance of the GI subcategory slope was also performed with experimental group and sex used as predictors. This analysis of variance found a significant relationship with experimental group ($F(2,36)=4.05,p=0.02$), as well as participant sex ($F(1,36)=5.11,p=0.03$). Here, a Kruskal-Wallis test did identify a statistically significant impact of experimental group on GI slope (${\chi }^{2}\left(2\right)=9.34,p=0.009$). This was followed by a series of pairwise Wilcoxon Rank Sum tests with a Bonferroni correction, which found that the GI slope of the anticipatory cues group was significantly lower than both the control group ($W(11)=143,p=0.03,f=0.80$) and the active posture group ($W(11)=146.5,p=0.02,f=0.81$) with large effect sizes.

### Nausea and survival analysis

**Fig. 4:**
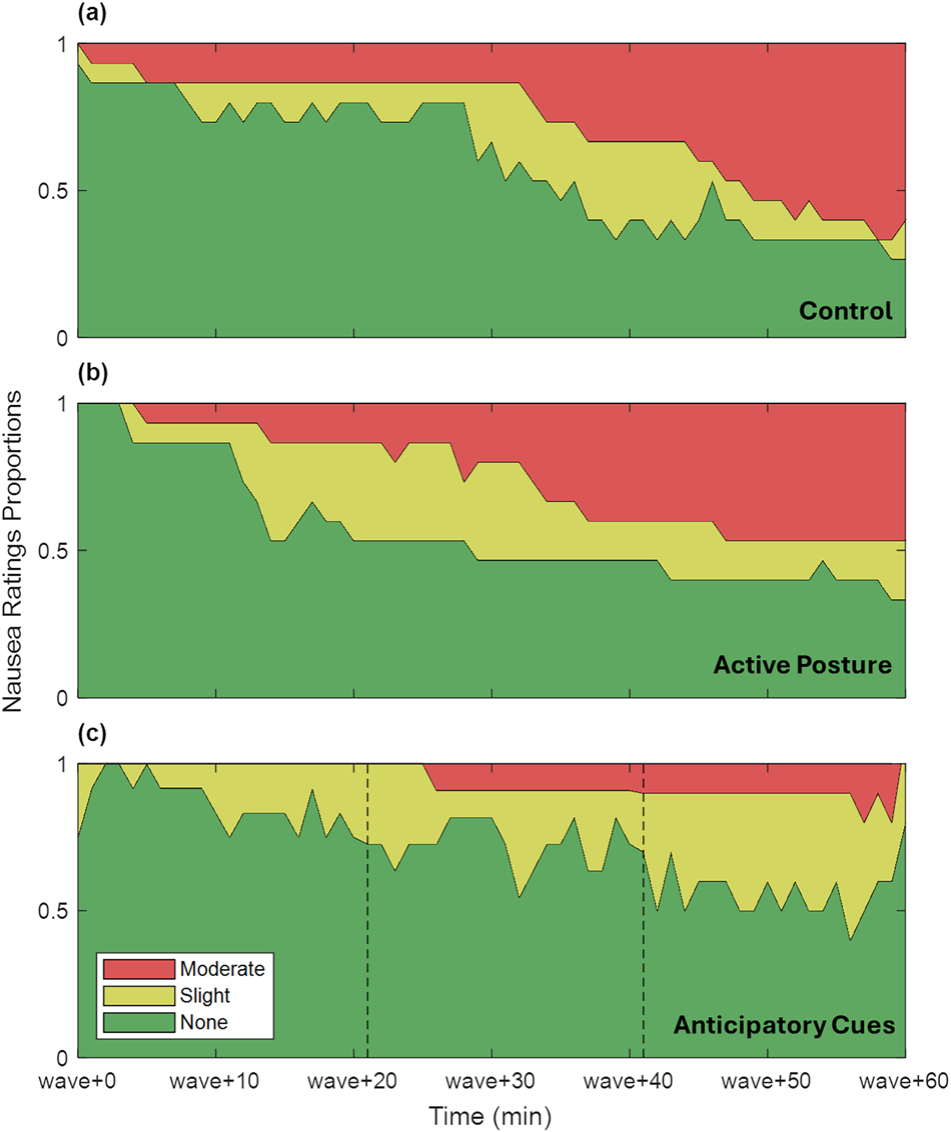
Nausea level proportions during wave-like motion. Levels for the **a** control group, **b** active posture group, and **c** anticipatory cueing group are presented. Red (top color) is for ‘moderate’ nausea, yellow (middle color) is for ‘slight’ nausea, and green (bottom color) is for no nausea. Subjects that dropped out are held at ‘moderate’ nausea. Dropouts due to technical issues are noted with vertical dashed lines.

To provide a more frequent motion sickness measure that took less time than the MSQ, every minute, subjects also reported nausea on a scale of “none”, “slight”, “moderate”, or “severe” (Fig. 4). All three groups showed similar patterns where the proportion of slight and moderate nausea increased with time in wave-like motion, but the temporal dynamics varied slightly. In Fig. 4a, subjects in the control group had relatively low nausea until roughly ‘wave+30’ where the slope of development became steeper for both the slight and moderate nausea. Conversely, the slope in Fig. 4 for the active posture group (Fig. 4b) was more constant from the beginning of motion. Finally, subjects in the anticipatory cues group (Fig. 4c) also experienced nausea, but development halted at ‘slight’ nausea for most of the subjects rather than progressing to ‘moderate’ as in the other two experimental groups (Fig. 4c).

To prevent subjects from reaching emesis, a stopping criterion was predefined in which wave-like motion was halted prematurely if a subject reported two consecutive minutes of moderate nausea, which is a useful endpoint equivalent to a non-transitory experience of being halfway to the point of vomiting^51^. Pre-vomiting experimental endpoints have been employed in many studies^47^, and avoid cases of sudden vomiting, while ensuring that subjects are experiencing functionally significant levels of discomfort. In our survival analysis, subjects who were able to withstand 60 minutes of wave-like motion were considered ‘survivors,’ while those who needed to stop wave-like motion early due to sustained moderate nausea were classified as ‘non-survivors.’

**Fig. 5:**
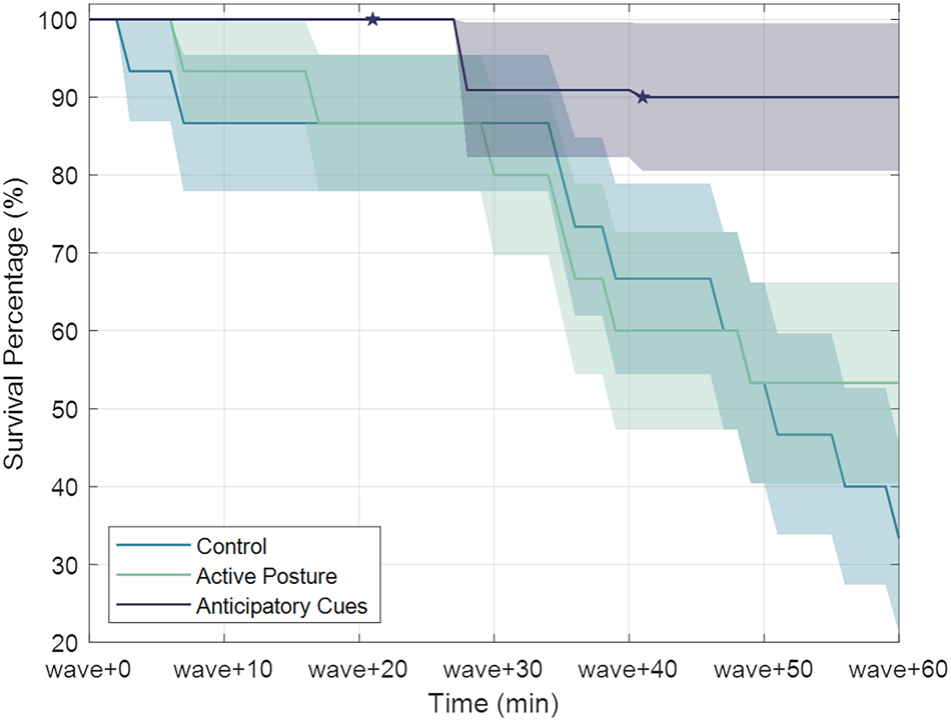
Survival percentages for each experimental group with standard error. Stars indicate dropouts due to technical issues, reducing sample size.

Figure 5 shows the percentage of subjects that had not dropped out by various times during wave-like motion (with shaded regions depicting standard error of the proportions). By the end of the 60 minutes, 33% of control group subjects and 53% of the active posture group survived without reaching sustained moderate nausea, compared to 90% of the anticipatory cues. As expected from the nausea reports shown in Fig. 4, there was a sharp decline in survival around ‘wave+30’ for the control group, a steady dropout rate for the active posture group, and barely any dropout in the anticipatory cues group, since nausea rarely progressed past ‘slight’.

A binomial generalized linear model assessed survival rate at ‘wave+60’ as a function of experimental group, participant MSSQ percentile, and participant sex. There was a significant effect of the anticipatory cues countermeasure ($\beta =4.31,p=0.004$), as well as a statistically significant ($\beta =2.80,p=0.01$) effect of sex where male subjects were more likely to survive. A post-hoc test of proportions with a Yates Continuity correction was performed between the anticipatory cues countermeasure and the control group which found a significantly higher survival rate in the anticipatory cues group (${\chi }^{2}(1)=5.67,p=0.017$). A measure of effect size was performed using Cohen’s h and found a large effect from this countermeasure ($h=1.27$).

Survival of the active posture group was separately analyzed as a function of postural performance and strategy during the wave-like motion. Head motion was tracked using an inertial measurement unit (IMU). Recall that during wave-like motion, subjects in the active posture group were instructed to use their head and torso to keep their head upright with the Earth vertical. The Earth vertical was used in the instructions as opposed to “sensed vertical”^52^ for simplicity to avoid the need for training subjects in the difference between the two. Subjects accurately performing the given task should have had a roughly zero and constant roll tilt angle; however, it is possible that subjects could have alternatively aligned themselves with the net gravito-inertial force (GIF) – the combination of gravity and the force from linear acceleration (i.e., the sensed vertical) – misconstruing it for true vertical. We determined this was not the case. The postural performance metric for postural control was the root mean squared (RMS) of the roll tilt angle, where lower values represented better performance. We calculated the root mean squared error (RMSE) between the roll tilt angle and the GIF angle relative to Earth-fixed vertical as well. Here, a smaller value implies subjects were aligning with the GIF rather than gravity.

**Fig. 6:**
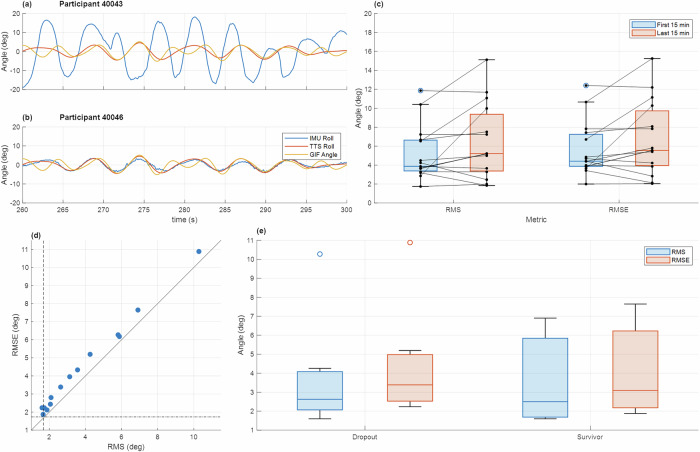
IMU metric visualization during active posture countermeasure. In **a** and **b**, head tilt is shown in blue against the roll of the motion device (red) and the angle of the net gravito-inertial vector (yellow) for two participants. In **c**, both performance metrics are shown for the first 15 minutes (blue) and the last 15 minutes (red). A comparison of RMS and RMSE values is shown in (**d**) with a unity line plotted. The dashed lines indicate the RMS and RMSE of the tilt profile (no head movement). Performance metrics grouped by subject survival is shown in (**e**) with RMS in blue and RMSE in red.

Examples of subjects performing the task are shown in Fig. 6. Some subjects seemed to arbitrarily tilt their head side to side (Fig. 6a) while others did not appear to move their heads at all (Fig. 6b). Performance metrics were calculated during each 15-minute segment of wave-like motion to determine if subjects’ performance changed throughout the motion. From the first segment to the last segment, no significant changes in performance were detected (Fig. 6c, Wilcoxon Signed Rank: $W(14)=24,p=0.076$), so the performance metrics were calculated over the entire wave-like motion.

Two Wilcoxon Rank Sum tests were performed to determine if the surviving subjects had a significantly different RMS or RMSE than the dropouts (Fig. 6e), but no significance was found ($W{\left(14\right)}_{{RMS}}=181.5,{p}_{{RMS}}=0.81{;W}{\left(14\right)}_{{RMSE}}=178.5,{p}_{{RMSE}}=0.56$). Additional Pearson correlation tests were performed to determine if there were correlations between these active posture metrics and the development of motion sickness symptoms via the MSQ and MSQ subcategory slopes, but again, no significant relationships were found ($p\in [0.53\mbox{,}\,0.98]$).

### Balance

Vestibular-mediated balance was assessed at three points throughout the experiment using the modified Romberg balance test condition 4 (with eyes closed and on a three-inch thick medium density foam pad^53,54^: 1) prior to the 2Gx centrifugation gravity transition analog (pre-SIC), 2) immediately after the wave-like motion (post-wave) and 3) following an additional hour of recovery (post-recovery). At each time point, eight trials of up to 30 seconds were performed. If balance was not lost, it was counted as a “passed” trial. Sway was recorded with an IMU mounted on the subject’s lower back.

**Fig. 7:**
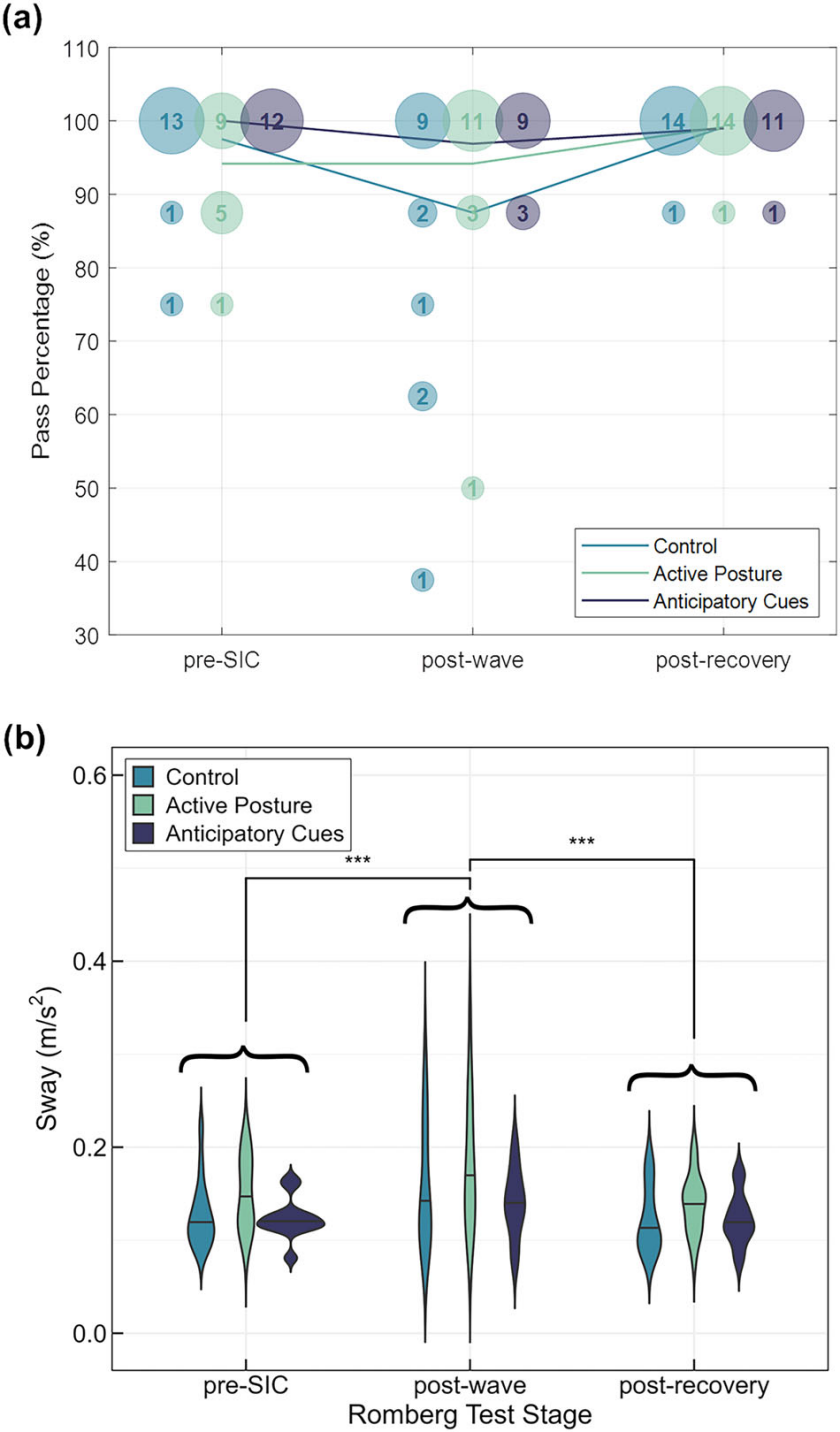
Modified Romberg Balance Test results. **a** shows the percent of successfully completed trials at each experimental timepoint while **b** shows the root mean square off-axis sway for each experimental group at each timepoint.

The results from the modified Romberg balance tests are shown in Fig. 7. Figure 7a shows how many subjects in each experimental group were able to complete a given percentage of the eight balance trials at each time point. The bubbles represent the number of subjects able to complete each percentage of tests, while the lines show the average percentage of completed trials across subjects. For the control group and the anticipatory cues group, there appeared to be a decline in performance for many subjects following wave-like motion that effectively resolved itself by the end of the recovery period. Meanwhile, for the active posture group, performance seemed to improve following wave-like motion and even more so after recovery.

Considering a separate metric of performance during balance tasks, off-axis sway is presented in Fig. 7b through a series of violin plots. We performed an aligned rank transform analysis of variance on the sway data to determine if time point or experimental group had a significant impact on sway. This model fit revealed a significant effect of time point ($p=2.1\times {10}^{-7}$), but not experimental group. Post-hoc Wilcoxon Signed Rank tests with a Bonferroni correction identified a significant difference in sway between the ‘pre-SIC’ and ‘post-wave’ time points ($W\left(41\right)=759,p=0.0002,r=0.84$), and the ‘post-wave’ and ‘post-recovery’ time points ($W\left(41\right)=849,p=6.9\times {10}^{-8},r=0.94$). Effect sizes for these tests are reported using the rank-biserial correlation, $r$. These results imply that balance performance across all three groups declines immediately following the wave-like motion before returning to baseline performance after recovery.

## Discussion

The purpose of this study was to assess if approaches aimed at improving the accuracy of sensory expectations would reduce motion sickness incidence and severity in a simulated post-spaceflight water landing^55^. Concerning the efficacy of active postural control and anticipatory cues as countermeasures during reentry, our results revealed that providing visual anticipatory cues significantly reduces the progression of gastrointestinal disturbance symptoms, as evidenced by the slope of the GI subcategory of the MSQ during wave-like motion (see Fig. 2a). This finding is bolstered by the anticipatory cues group having significantly better survivability (i.e., no consecutive reports of moderate nausea) during wave-like motion. Specifically, the anticipatory cues group had nearly triple the survival rate of the control group at the end of an hour of wave-like motion (90% vs. 33%). Within the context of sensory conflict theory, this result implies that veridical visual information about upcoming motions helps the brain formulate a more accurate expectation of future incoming sensorimotor information. When compared to visual cues without anticipatory information (see^40^, 78% survival, see Supplementary Figure 1), the survival rate was still higher (albeit without a statistically significant difference: $p=0.85$), suggesting that the addition of anticipatory information may help improve survivability.

This study marks the first instance of using anticipatory visual cues or active posture in the context of a simulated post-spaceflight water landing. Previous studies utilizing anticipatory cues typically consisted of low-fidelity visuals such as light panels or LED strips^30–32^ to communicate about specific upcoming maneuvers and were only used in standard terrestrial forms of travel. Our anticipatory cues provide information about future orientation and acceleration intuitively (including the direction and magnitude of the net gravito-inertial force vector), and the signals are incorporated into a visual scene showing cues of current motion. The results of the study – that anticipatory cues are effective at reducing the incidence and progression of nausea and nausea-related symptoms – imply that providing cues of upcoming motion to crewmembers within a capsule would be an effective strategy to reduce the incidence and severity of EMS. However, the most effective modality and predictive timing would still need to be investigated, as well as the benefits of anticipatory cues without the visual scene.

In contrast, we found active postural control to be insufficient in attenuating the development of motion sickness symptoms during our EMS paradigm compared to the control condition. While we had hypothesized that encouraging active postural control would enable better sensory expectations, thereby reducing sensory conflict and helping mitigate motion sickness, there are mixed opinions in the literature. In a study performed by Mills and Griffin^56^, subjects exposed to sinusoidal horizontal translations experienced more motion sickness in the absence of upper body restraints than those with restraints, and in a study evaluating the incidence of airsickness in over 500 airmen, Johnson and Mayne^57^ found head restraints to be effective at significantly reducing sickness during flight across all examined flight environments compared to a control that allowed participants to move their heads freely. Notably, these studies did not report that subjects were instructed to keep their heads “upright” when unrestrained, as we did in the active posture countermeasure group. However, our subjects were generally unable to follow the instructions during wave-like motion. Further, we posit that the underlying neural mechanisms that can produce this reafference cancellation during active head motions, which is typically beneficial when commanding motion, often become detrimental during active motion superimposed on a passive trajectory without reliable cues of motion.

Previous reports have found that active head movements, with visual or other non-vestibular fiduciary information, accelerate adaptation following gravity transitions^58,59^. This would have been most notable for the active posture group; however, investigating our secondary metrics, we did not detect any differences between balance performance across experimental groups. This may have been caused, in part, by the variable exposure time to wave-like motion due to our stopping criteria or the lack of veridical visual information to facilitate adaptation. Instead, there was a significant decline in balance performance following centrifugation and wave-like motion across all three experimental groups. RMS of linear acceleration in the inertial XY-plane was significantly higher during the post-wave stage than both the pre-SIC and post-recovery stages. This validates our ground-based analogs for some sensorimotor decrements relevant to post-spaceflight environments.

Across all experimental groups, we found evidence correlating motion sickness and anxiety with the MSQ and STAI metrics as suggested by previous studies^42,47,60^. Additionally, our analyses revealed that female subjects in general reported on more motion sickness than male subjects as has been reported by some previous studies^61–64^ but questioned by others^65,66^.

**Fig. 8:**
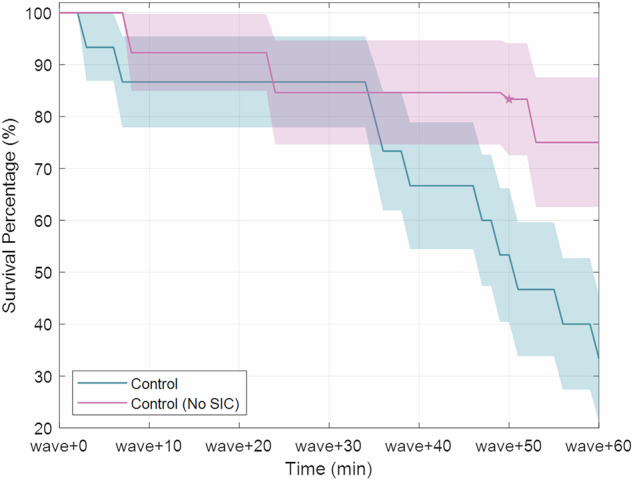
Survival comparison between control groups. Control group with and without Sickness Induced by Centrifugation presented with standard errors.

We used two ground-based analogs to mimic the effects of a gravity transition followed by sea state motion: Sickness Induced by Centrifugation (SIC), followed by wave-like motion using roll tilt and lateral translation. The efficacy of SIC as an analog for gravity transitions has been characterized in Nooij and Bos^55^ for net forcings of 2G and 3G at 45 minutes and 90 minutes. The strongest motion sickness effects were seen at the highest and longest centrifugation regimes. To characterize our 56-minute 2G profile, we collected an additional subject cohort that experienced the same wave-like motion as the control group without centrifugation. This “No SIC” group (*n* = 13, MSSQ = 38.4 ± 26.0%) had a survival rate of 75% in comparison to the 33% survival rate of the control group that experienced SIC (Fig. 8), suggesting that SIC may have worsened the nausea experienced by participants during wave-like motion (${\chi }^{2}\left(1\right)=3.12,p=0.077,h=0.87$ with Yates correction, $p=0.031$ without). This was expected as SIC has historically produced head movement-contingent vertigo. When immediately followed by passive motion, this would result in increased motion sickness.

This study is limited by the leveraged paradigms. The SIC paradigm is used as an analog for gravity transitions, but the sensory reinterpretation experienced by astronauts following extended exposure in microgravity likely follows a different time course in both readaptation^67,68^ and motion sickness symptoms. Additionally, inter-subject susceptibility to SIC is both highly variable and not captured in the MSSQ. This may have resulted in experimental groups being imbalanced in SIC-susceptibility. To gauge a potential imbalance, MSQ scores just prior to wave-like motion were compared across groups, and no significant differences were detected. For the wave-like motion paradigm, the Tilt Translation Sled (TTS) cannot incorporate motion along the Earth-fixed vertical into the motion profile even though it is believed that the low-frequency heave aspect of a sea state is especially provocative^69^.

The relatively high drop-out rate in certain subject groups is an additional study limitation of this study, and one that is encountered frequently in motion sickness research. This required careful extrapolation and analysis of the remaining data. While our linear extrapolation poses its own limitations, removing subjects who reached the stopping criteria would have resulted in MSQ and STAI data skewed (substantially) towards subjects with low SIC susceptibility, potentially obscuring any impact of the countermeasures. Instead, we employed the linear extrapolation as a proxy for motion sickness that may have been experienced by those subjects who dropped out, but remained robust to the exact extrapolation by using medians for data visualization and non-parametric statistics for analysis. We believe this was a conservative approach and are encouraged that, despite the limitation of our extrapolations, none of our significant results explicitly rely on any extrapolated data.

Providing accurate visual cues and anticipatory cues within an operational environment is another challenge to consider. We were able to provide accurate cues of present self-motion and future motion as the wave-like motion profile was known ex-ante. In an operational environment, an IMU could be used to determine the present orientation state, as has been done in cars previously by Hock et al.^70^. However, these works have presented visual information to subjects seated upright. The visual information for a reclined astronaut within a capsule will need to be presented differently. Providing cues of future motion also poses a challenge as it may require extrapolating into the near future, and further work is needed to define how accurately that must be done to remain effective. Additionally, the prediction time should be similarly characterized to establish the minimum time in the future needed to reduce the effects of motion sickness.

An avenue of future investigation could entail determining whether veridical information is the most beneficial sensory input to provide. Following gravity transitions, the expected afference may differ from afference generated by ground-truth motion. In place of veridical information, computational models of motion sickness^71^ could be leveraged to identify sensory inputs that would reduce sensory conflict post-transition. These inputs could include visual information such as real-time cues of self-motion or anticipatory cues, or alignment guides for active posture. Further, sensory conflicts are believed to elicit recalibration of neural expectations via sensory reinterpretation^72–74^ and reweighting^67,75^. Thus, in the absence of manipulating sensory cues, sensory conflict is self-limiting: gravity transitions evoke it, and its prolonged presence drives recalibration that eliminates it. However, until recalibration is complete, sensory conflicts that exceed thresholds activate emetic response pathways within the central nervous system (CNS)^24,76^. Thus, the ideal sensory cue manipulations would balance attenuating transitory sensory conflicts enough to ameliorate motion sickness but not enough to prevent the recalibration of neural expectations.

Another avenue of future investigation is the effectiveness of active posture in reducing motion sickness with visual information available. If the vestibular system is unreliable following a gravity transition, the addition of visual cues of self-motion would allow subjects to more precisely move their head to maintain upright. The visual information for this task may not need to be as rich as the one provided in this study, but could be closer to an artificial horizon as provided in previous studies^77^.

## Methods

**Fig. 9:**
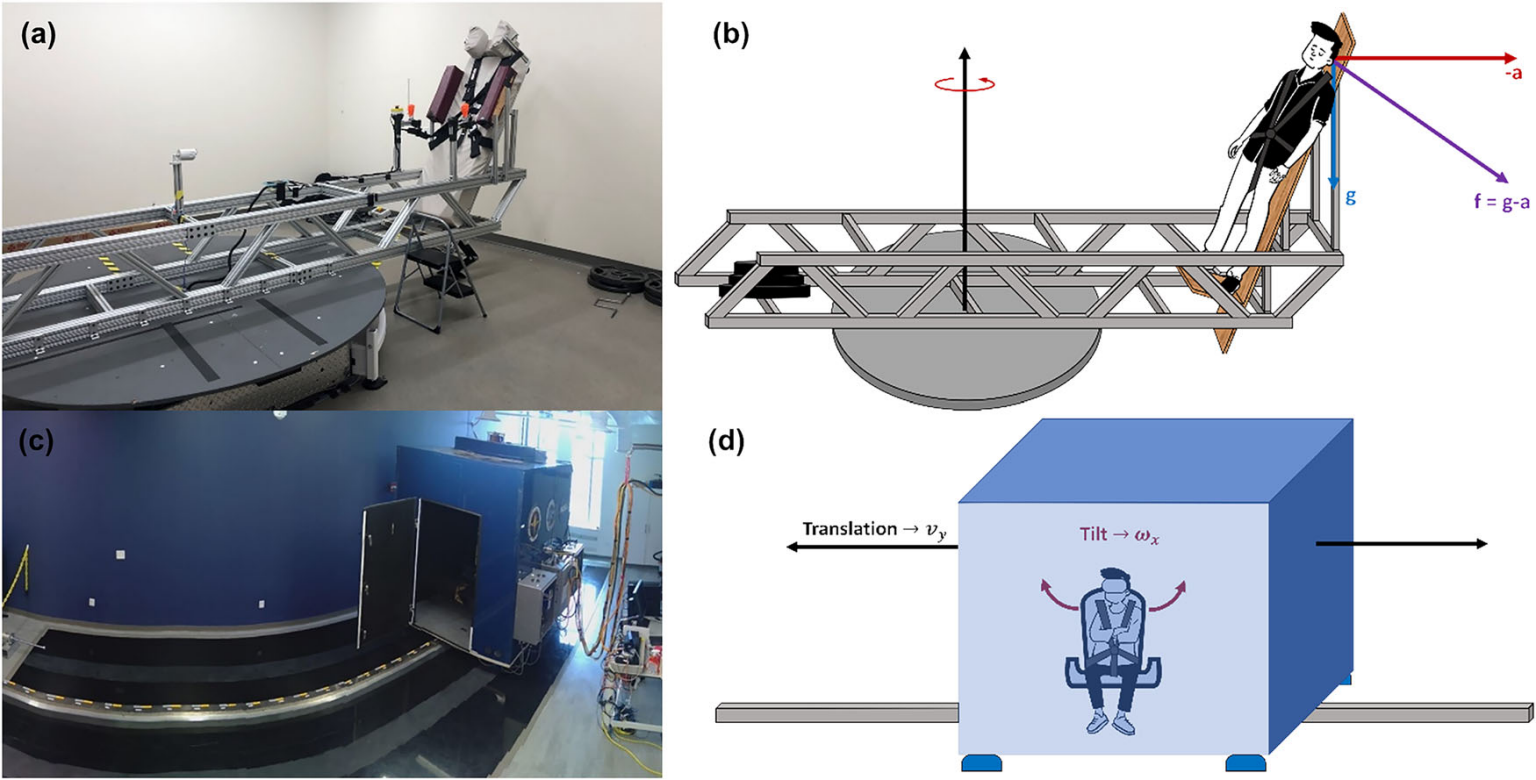
The motion devices used for simulating a post-spaceflight water landing. The centrifuge (**a**, **b**) rotated at 24 RPM to generate a net 2 g’s at the head while the sled (**c**, **d**) produced roll tilts and lateral translations at the frequencies of an ocean buoy. Note that the curvature in the track in (**c**) is a result of a fisheye lens. The track is straight.

To assess the efficacy of the selected countermeasures in reducing the incidence and severity of motion sickness in astronauts following a water landing, we utilized a series of ground-based analogs for the gravity transition and sea state motion astronauts experience. Protocols and motion devices were identical to those we have used previously in Lonner et al.^40^. Gravity transitions were simulated on the Human Eccentric Rotator Device (HERD, Fig. 9a, b) centrifuge using the Sickness Induced by Centrifugation (SIC) paradigm^39,78–80^: exposing subjects to a net 2Gx gravito-inertial force for approximately one hour to generate a vestibular disturbance upon return to Earth gravity. Immediately following, the ocean state was simulated in the Tilt Translation Sled (TTS, Fig. 9c, d) using roll-tilt and lateral translation to generate a wave-like motion profile that subjects experienced for up to an hour. Visual cues for all experimental groups were generated and controlled through the game-engine development platform Unity and were delivered through a Meta Quest 2 virtual reality (VR) headset.

During wave-like motion and the subsequent hour of stationary recovery, the progression of motion sickness symptoms and anxiety was recorded using subjective verbal reports. For subjects performing the active posture condition, a head-mounted inertial measurement unit (IMU) was used to record subjects’ techniques and performance during the task. Standing balance was assessed as a measure of sensorimotor performance through a modified Romberg balance test at three points during the experiment: prior to centrifugation, following wave-like motion, and after recovery. An IMU was used to record sway during these tests.

### Sickness induced by centrifugation

The SIC paradigm posits that extended exposure to hyper-gravity followed by a return to Earth gravity recreates many of the challenges experienced by astronauts undergoing gravity transitions such as motion sickness, postural instabilities, and gait destabilization^2,39,55,78,80–84^. Pro-longed centrifugation is theorized to cause sensory re-interpretation within the graviceptors due to the presence of a constant modified GIF. As such, SIC has been used in previous studies as a ground-based analog for both Space Motion Sickness (SMS) when astronauts first enter microgravity^78,85,86^, as well as Entry Motion Sickness (EMS) when astronauts return to Earth after adaptation to microgravity^40^. This includes the presence of head movement-contingent vertigo and self-motion illusions that are believed to worsen motion sickness in response to head tilts^86^. The severity of symptoms following SIC depends on the magnitude and duration of centrifugation, with higher and longer hyper-gravity exposure resulting in more severe symptoms and a slower recovery time^55^.

Subjects experienced centrifugation at 2Gx for 56 minutes plus spin-up and spin-down time. Spin-up took roughly six minutes, with some variation due to levels of subject comfort, while spin-down was done over two minutes. The duration and magnitude of hyper-gravity exposure used was expected to generate mild-to-moderate symptoms of SIC^55^. After subjects were spun down, they were helped out of the centrifuge and transferred into a wheelchair with their head restrained to discourage provocative head movements. The operator then wheeled the subject down the hallway to the TTS for the next portion of the experiment. This transfer window typically took less than eight minutes.

### Sea state simulator

To simulate the sea state astronauts are exposed to during water landings, a two degree of freedom (DOF) motion simulator was employed. The TTS (Fig. 9c, d) is a motion device capable of head-centered roll-tilt and lateral (Earth-horizontal) translation along a linear track. The wave-like motion profile developed for this experiment sampled the energy spectra from Coastal Data Information Program (CDIP) Station 067 to create a semi-coupled tilt-translation profile. CDIP-067 is a buoy located about 65 miles off the coast of Southern California, situated in a candidate site for future Orion landings. To recreate a realistic sea state for water landings, the buoy data was extracted during NASA’s Underway Recovery Test-7 (URT-7). At this time, the significant wave height was 1.02 meters on average, corresponding to a three – or slight sea state – on the WMO Sea State Code. The frequency content of the wave-like motion ranged from 0.055 Hz to 0.23 Hz. The wave-like motion used here was identical to that used previously and additional detail can be found there^40^.

Following centrifugation, subjects were assisted with sitting in the TTS chair. Subjects experienced up to an hour of wave-like motion – dependent on a pre-determined stopping criterion – followed by an hour of recovery where subjects sat on a stool across from the operators in the illuminated lab space. While in the TTS, subjects wore a Meta Quest 2 VR headset. The headset strap and lenses were adjusted for the subject prior to centrifugation. To properly set the lenses, inter-pupillary distance (IPD) was measured using either EyeMeasure (iOS) or GlassedOn (Android), and the closest lens setting available on the Quest 2 was used.

During both the HERD and TTS protocols, two-way audio communication was available between the subject and the operators. Additionally, infrared video was used to monitor the subject, and both the subject and the operators had mechanisms available to halt motion.

### Independent variable

Two different methods for generating expectancy cues were assessed in their efficacy to reduce the incidence and severity of motion sickness when compared to a previously-collected control group in a between-subject design^40^. The first experimental group utilized active postural control wherein the subject’s head and torso remained unrestrained (only secured with a lap belt) while experiencing wave-like motion, and they were instructed to use their “neck and torso to keep their head upright with Earth vertical”. This implied that a positive (right ear down) passive chair roll tilt should be countered with a negative (left ear down) active head/torso roll tilt such that the net tilt is zero (i.e., head remained near upright). While performing this task, subjects wore a Meta Quest 2 VR headset that displayed a head-fixed white dot on a black background, providing no cues of self-motion in tilt or translation.

**Fig. 10:**
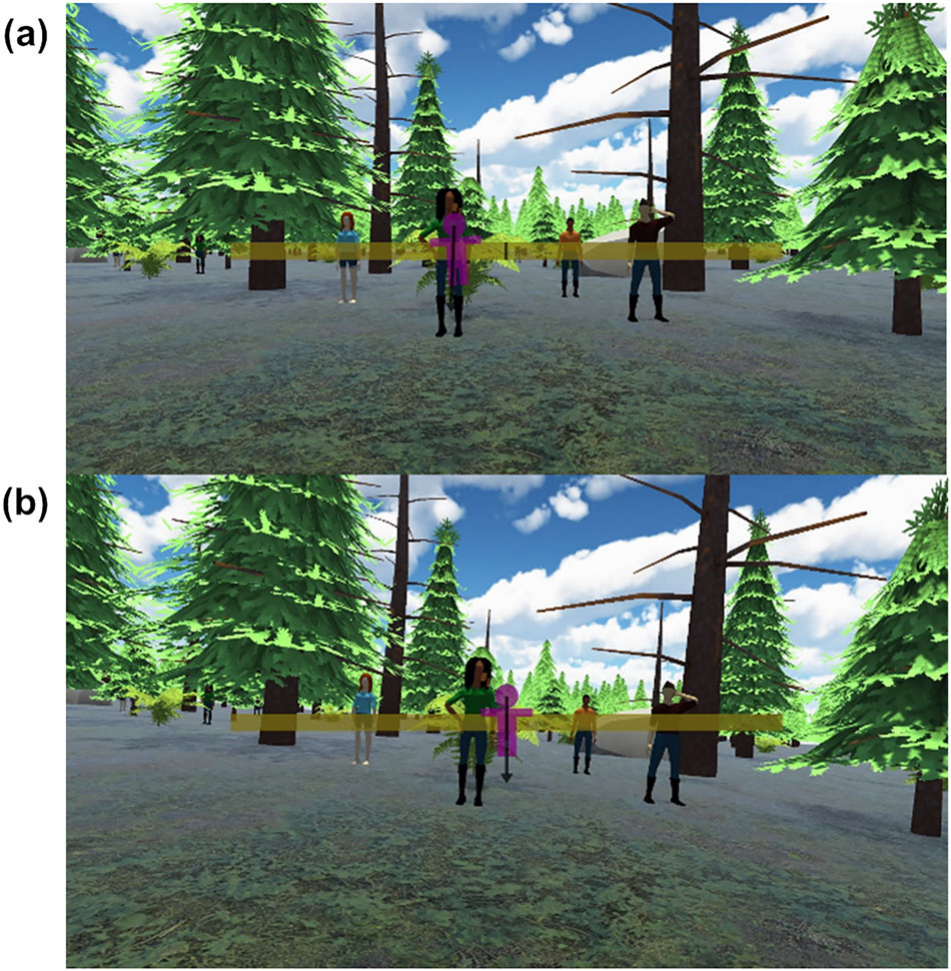
Anticipatory cueing visual scene. **a** demonstrates how the overlay shows future movement while **b** shows how the scene moves in response to a physical motion.

The second experimental group received both real-time and anticipatory visual cues through the VR headset. Exactly as we tested previously^40^, real-time cues were delivered through an Earth-fixed rich forest scene that moved opposite the direction of subject movement to provide veridical cues of self-motion. As a novel addition to the VR scene, anticipatory cues were also given by means of a semi-transparent overlay in front of the forest scene that showed a magenta “gingerbread” figurine on a yellow track moving as the subject would one second in the future. The overlay was an “outside in” display where the figurine would tilt and translate along a head-fixed, yellow bar representative of the translation track. Finally, a black arrow depicted the direction and relative magnitude of the net gravito-inertial force (i.e., the combination of gravity and inertial acceleration, or that which the subject would feel being pulled one second in the future). We hypothesized that this would provide rich cues of tilt, translation, rotation, and the synthesized net gravito-inertial force that the subject could use to anticipate upcoming sensory stimulation to help mitigate motion sickness. Two example screenshots of these visuals are shown in Fig. 10. In this second experimental group (anticipatory cues), during the wave-like motion, subjects’ torsos were restrained with a five-point harness, and their heads remained aligned with their torsos by an adjustable, padded head restraint.

The control group results were presented originally by Lonner et al.^40^. The subjects in this control group experienced the same duration and magnitude of centrifugation, followed by the same wave-like motion profiles, but had their head and torsos restrained during wave-like motion, as in the present anticipatory cues group. They had the same head-fixed white dot displayed on the VR headset as the present experiment's active posture group. To minimize potential between-group confounds from using the VR headset, the screen brightness was fixed, and subjects were given an opportunity prior to testing to size the headset comfortably for themselves.

### Dependent variables

A variety of subjective and objective metrics were collected throughout this experiment to understand the impacts of the countermeasures on motion sickness, anxiety, and sensorimotor performance. Motion sickness was assessed using a Motion Sickness Questionnaire (MSQ)^41,42^ every five minutes during wave-like motion and recovery. This multi-symptomatic questionnaire covers 28 symptoms linked to motion sickness and has the subject rate each one on a scale of ‘none’, ‘slight’, ‘moderate’, or ‘severe’. These ratings can be converted to an ordinal score by converting the ratings to a 0–3 scale and taking the sum across the symptoms at each time point to get a temporal progression of motion sickness. The minimum MSQ score attainable is zero, while the maximum theoretical MSQ score is 84. However, since some of the symptoms are antithetical in nature (e.g., “increased salivation” and “decreased salivation”), it is unlikely that this score would ever be reached. Prior to centrifugation, subjects were briefed on all 28 of the symptoms they would be asked about during wave-like motion and recovery and given the opportunity to request definitions for any of the symptoms. The definitions provided were consistent with those available from Lawson, 2014^47^ supplemented with Oxford Languages 2022 for definitions not explicitly defined by Lawson.

To extract a more precise understanding of the subjects’ experiences, symptoms can be further separated into six categories outlined in Cha et al., ^44^: gastrointestinal disturbance, thermoregulatory disruption, alterations in arousal, dizziness/vertigo, ocular, and general. In considering the symptom categories most likely to impair astronauts during post-spaceflight water landings, this study investigated the variations in gastrointestinal disturbance (GI) and motion-related alterations in arousal (sopite) between experimental groups. Additionally, since VR headsets were being utilized during the experiment, ocular symptoms (e.g., headache, blurred vision, difficulty focusing) were also considered. The scoring of these subcategories was performed by summing 0-3 ‘none’ to ‘severe’ scores on each relevant symptom rating, as in the full MSQ score.

In addition to administering the MSQ every five minutes, a two-symptom subset of the MSQ was assessed every minute during wave-like motion and recovery, to gauge subject’s “nausea” and “general discomfort”. This metric was used to track subject well-being. To prevent subjects from reaching emesis, a stopping criterion was predefined, in which wave-like motion was halted prematurely if a subject reported two consecutive minutes of moderate nausea.

Both motion sickness and anxiety (in the absence of motion) can elicit nausea. Furthermore, subjects exposed to unusual motion situations might be expected to experience some anxiety^40,60^. For these reasons, we tracked the potential anxiety confound throughout wave-like motion and recovery using a modified State-Trait Anxiety Inventory (STAI)^43^. The modified STAI is a six-question survey that asks subjects to rate declarative statements about their present emotional state on a scale of ‘not at all’, ‘somewhat’, ‘moderately so’, or ‘very much so’^43^. This modified STAI focuses on the state aspect of a subject’s anxiety, meaning their anxiety at a given time rather than characteristic trait anxiety. Scoring of the STAI is done on a 1–4 scale where a higher number means the subject is experiencing more anxiety. In the cases of positive declarations such as “I feel calm”, the highest value is associated with a response of “not at all”, while for negative declarations like “I feel tense”, the highest value is associated with a response of “very much so”. Therefore, the minimum possible score for the STAI is 6, while the maximum score is 24. The STAI was asked in the same five-minute intervals as the MSQ.

Objective measures were also recorded to investigate the impact of our experimental analogs on sensorimotor performance as a secondary metric. At three points throughout the experiment, subjects performed a modified Romberg balance task (‘condition 4’ for isolating vestibular-mediated contributions)^53,54^, which required the subjects to balance on a foam pad with their feet together, arms crossed, and eyes closed for eight 30-second trials. Failure criteria included a subject opening their eyes, separating their feet, uncrossing their arms, or otherwise losing balance, requiring intervention from test conductors. This task was performed prior to centrifugation to collect a baseline performance, after wave-like motion, and after the recovery period. The number of successfully completed 30-second trials (out of 8 attempts) at each time point was documented, as well as accelerometer and gyroscope outputs using an IMU secured to the center lower back and sampling at a rate of 25 Hz. The sway metric generated for this experiment was the median root mean squared (RMS) of linear accelerations in the inertial XY-plane (perpendicular to gravity) between the eight trials. To reduce the impact of learning effects, subjects were given three easier practice trials prior to their first set of eight modified Romberg balance tasks. For these 30-second trials, subjects were tasked with balancing on solid ground with their eyes open, eyes closed, and on the foam pad with their eyes open.

Finally, to quantify active postural activity during wave-like motion in that countermeasure group, a head-mounted IMU tracked linear acceleration and angular velocity at a sampling rate of 30 Hz. This informed how well subjects were able to perform the task of attempting to keep their head upright during the motion, and if different techniques resulted in differences in incidence and severity of motion sickness.

### Experimental design

This study used a between-subject design where the experimental groups were balanced by motion sickness susceptibility and sex. The Short-Form Motion Sickness Susceptibility Questionnaire (MSSQ)^48,49^ was used to predict subject susceptibility to terrestrial motion sickness based on past experiences^58,87,88^. Experimental groups were block-sorted such that the MSSQ percentile mean and standard deviation were comparable as well as the proportion of female subjects. Subjects with an MSSQ score in the outer 5th percentiles were excluded from the study to pre-emptively remove potential outliers from individuals who are highly susceptible or not prone to motion sickness. Historically, the MSSQ has not been used as a predictive measure for motion sickness caused by gravity transitions, but recent work correlating MSSQ with motion sickness during parabolic flights^89^ suggests that it may have merit as a predictor for SMS or EMS.

Subject recruitment was done primarily through flyers posted within the Aerospace Building at the University of Colorado, Boulder, yielding a cohort of predominantly college-aged students. As such, the ages of the experimental groups balanced naturally.

### Participants

**Fig. 11:**
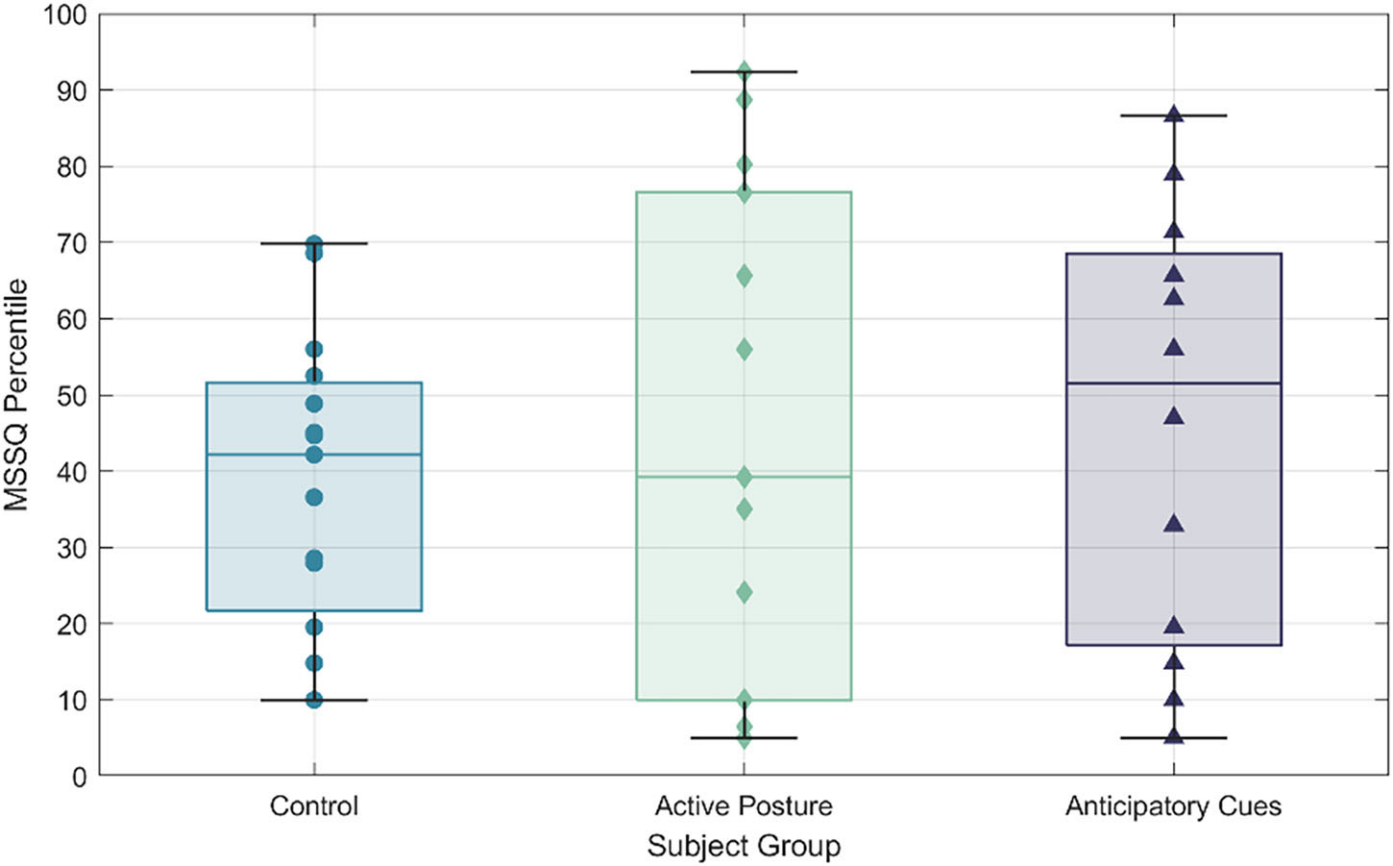
MSSQ spread between experimental groups. Individuals within the control group (blue) are denoted by circles, those in the active posture group (green) are denoted by diamonds, and subjects in the anticipatory cues group (purple) are denoted with triangles.

The study was approved by the Institutional Review Board of the University of Colorado (FWA00003492) in accordance with the Declaration of Helsinki. All participants signed a written informed consent prior to participating in the study. There was a total of 42 participants across the three experimental groups (age = 23.7 ± 4.7 years, female = 19, MSSQ = 42.8 ± 27.2%) with 15 subjects in the control group and the active posture group, and 12 subjects in the anticipatory cues group. The control group (age = 23.2 ± 3.6 years, female = 5, MSSQ = 38.3 ± 19.5%) was presented previously in Lonner et al.^40^. The active posture group (age = 24.9 ± 6.2 years, female = 7, MSSQ = 44.7 ± 33.4%) had the same fixation point visual as the control group but had their head and torso unrestrained. The anticipatory cues group (age = 22.8 ± 2.7 years, female = 7, MSSQ = 45.9 ± 28.5%) was provided accurate present visual cues, as well as cues for their upcoming motion. The spread of MSSQ percentiles across all three groups is presented in Fig. 11.

A one-sided Wilcoxon Rank Sum test found that female subjects had statistically higher MSSQ (an assessment of past history of motion sickness^40^) percentile scores than male subjects ($U=293,p=0.03$), but a Kruskal-Wallis test was performed on the MSSQ percentiles for the three experimental groups and found no significant differences, so we assumed that all three groups were sampled from the same population (${\chi }^{2}\left(2\right)=0.43,p=0.81$), regardless of sex differences. Additionally, Kruskal-Wallis tests were performed for the STAI and MSQ scores prior to wave-like motion to determine if any of the groups were pre-disposed to anxiety of motion sickness. Neither test found significant differences.

### Data analysis

Multiple subjects were unable to complete the hour of wave-like motion either due to reaching the stopping criteria or experiencing technical difficulties with the motion devices. These dropouts can yield a biased sample among the surviving subjects, artificially lowering the average scores of the groups. As we have done previously^40^, we accounted for missing subject data (MSQ and STAI) by linearly extrapolating from data available during wave-like motion prior to dropout. If a subject only had one data point during wave-like motion, the first point during recovery was used along with the time spent experiencing wave-like motion to generate a conservative extrapolation. If the extrapolation reached the maximum of the scale (e.g., 24 for STAI), it was capped at that value. Since subjects continued directly into recovery following dropout, their final data point during wave-like motion was carried over to the ‘recovery+0’ time point. Extrapolated data is denoted by open shapes and dotted lines in figures. To maintain robustness against these extrapolations, median values were used for visualization as well as non-parametric statistics. MATLAB R2023b (The Mathworks, Inc.) and R 4.3.0 (RStudio 2023.03.0 + 386) were used to perform statistical analyses.

MSQ total and MSQ subcategory scores were compared across the three groups at the ‘wave+30’ and ‘wave+55’ time points using a series of cumulative link regression models to account for our ordinal, non-normally distributed data. These time points were selected as they are approximations of the times a capsule nominally would be captured by a recovery vessel and the time the last astronaut may be removed from the capsule following recovery^50^. For the full MSQ score, model predictors included MSSQ scores, experimental group, and STAI scores. The subcategory models only considered MSSQ scores and the experimental group.

In addition to the cumulative link models, the time progression of motion sickness was analyzed utilizing the linear slope of the MSQ during wave-like motion^90^. Since this is the same slope used to produce extrapolated data for subjects that dropped out, this metric is robust to subject dropout. An aligned rank transform (ART) model was used to identify any significant categorical predictors for MSQ or MSQ subcategory slopes, such as experimental group, high or low MSSQ, or other available demographic information. Additional ART models were used to identify significant factors impacting the sway of our subjects during the modified Romberg balance tasks.

Finally, postural performance and strategy for subjects in the active posture group were analyzed via the head-mounted IMU worn during wave-like motion. Postural performance refers to how well subjects were able to cancel the tilt of the motion device to remain upright with Earth vertical, while postural strategy refers to the methods subjects used that improved or worsened motion sickness. The data streams of TTS wave-like motion and the head-mounted IMU recording were time-aligned using the dominant linear acceleration in the lateral direction, which, upon visual inspection, yielded reliable alignment.

## Supplementary information

Supplementary Material

Supplementary Video 1

## Data Availability

Additional datasets generated during and/or analyzed during the current study are available on Open Science Framework (OSF) at the following link: https://osf.io/6nk4s/?view_only=2f6a96ecc9b14740880a29846bf13f63.
