# Supplementary material for: Reducing motion sickness during simulated astronaut post-spaceflight water landings using anticipatory cues or postural control
Source: NPJ Microgravity. 2025 Jun 2;11:21. doi: 10.1038/s41526-025-00478-9 (PMC12130277; doi:10.1038/s41526-025-00478-9)
Supplement: Supplementary file 1 — Supplementary Material [file 41526_2025_478_MOESM1_ESM.docx]

Supplementary Material:

**Supplementary Figure 1**

Supplementary Figure 1. Survival of Control and Visual Countermeasure groups from Lonner et al., 2023 in comparison with Anticipatory Cues experimental group from present manuscript.

**Supplementary Movie 1**

The provided movie shows how the anticipatory visual cues are overlayed on cues of present self-motions during passive motion in the Tilt Translation Sled. The stick figure tilts and translates one second ahead of the device’s motion.
